# Effects of temperature on physiological performance and behavioral thermoregulation in an invasive fish, the round goby

**DOI:** 10.1242/jeb.237669

**Published:** 2021-01-12

**Authors:** Emil A. F. Christensen, Tommy Norin, Iren Tabak, Mikael van Deurs, Jane W. Behrens

**Affiliations:** Section for Marine Living Resources, DTU Aqua: National Institute of Aquatic Resources, Technical University of Denmark, Kemitorvet, Building 202, 2800 Kgs. Lyngby, Denmark

**Keywords:** Aerobic scope, Climate change, Critical thermal maximum, Metabolic rate, *Neogobius melanostomus*, Temperature preference

## Abstract

Invasive species exert negative impacts on biodiversity and ecosystems on a global scale, which may be enhanced in the future by climate change. Knowledge of how invasive species respond physiologically and behaviorally to novel and changing environments can improve our understanding of which traits enable the ecological success of these species, and potentially facilitate mitigation efforts. We examined the effects of acclimation to temperatures ranging from 5 to 28°C on aerobic metabolic rates, upper temperature tolerance (critical thermal maximum, CT_max_), as well as temperature preference (*T*_pref_) and avoidance (*T*_avoid_) of the round goby (*Neogobius melanostomus*), one of the most impactful invasive species in the world. We show that round goby maintained a high aerobic scope from 15 to 28°C; that is, the capacity to increase its aerobic metabolic rate above that of its maintenance metabolism remained high across a broad thermal range. Although CT_max_ increased relatively little with acclimation temperature compared with other species, *T*_pref_ and *T*_avoid_ were not affected by acclimation temperature at all, meaning that round goby maintained a large thermal safety margin (CT_max_−*T*_avoid_) across acclimation temperatures, indicating a high level of thermal resilience in this species. The unperturbed physiological performance and high thermal resilience were probably facilitated by high levels of phenotypic buffering, which can make species readily adaptable and ecologically competitive in novel and changing environments. We suggest that these physiological and behavioral traits could be common for invasive species, which would only increase their success under continued climate change.

## Abstract (precis)

**Summary:** Phenotypic buffering of physiology and behavior, coupled with large thermal safety margins, may be competitively advantageous for the invasive fish round goby, and enhance its invasive potential with climate change.

## INTRODUCTION

Globalization of commerce during the last few centuries has resulted in unintended anthropogenic movements of species between geographically distant areas (Mooney and Hobbs, 2000). Some of these species establish and thrive in the areas they have been introduced to, and become invasive by causing loss of biodiversity, altering ecosystem functioning, and changing the physical structure of habitats, including aquatic ecosystems (Vitousek et al., 1996; Mooney and Hobbs, 2000; Hobbs et al., 2006; Hellmann et al., 2008; Mainka and Howard, 2010; Simberloff, 2011; Katsanevakis et al., 2014). Across taxa and environments, it has been hypothesized that warming induced by anthropogenic climate change is further increasing the dispersal rates of invasive species, facilitated by a broad environmental tolerance and/or the ability to adapt rapidly to novel conditions (Mooney and Hobbs, 2000; Sakai et al., 2001; Stachowicz et al., 2002; Hellmann et al., 2008; Walther et al., 2009). Consequently, global warming may escalate range expansion of warm-tolerant invasive species in temperate climate zones and intensify negative ecosystem effects (Mooney and Hobbs, 2000; Hellmann et al., 2008; Mainka and Howard, 2010; Marras et al., 2015).

Environmental temperature directly affects the physiology and behavior of ectothermic organisms, including fish, and therefore largely dictates species distribution (Woodin et al., 2013; Teal et al., 2015). Understanding how physiology and behavior respond to environmental temperature is therefore a prerequisite for making model predictions of species distributions (Peterson, 2003; Evans et al., 2015; Urban et al., 2016). However, experimental studies investigating the effects of temperature on important physiological and behavioral traits are rare for invasive species, limiting further development of models of range expansion (Mooney and Hobbs, 2000; Hellmann et al., 2008; McCann et al., 2018).

Metabolic rate is a key physiological trait of all organisms and represents the pace at which resources obtained from the environment are converted into high-energy compounds (primarily ATP) that are used to sustain life and perform activities (Brown et al., 2004). Metabolic rates of fish have traditionally been partitioned into two extremes: the standard (resting) metabolic rate (SMR), which is the minimum level of metabolism an animal needs to maintain homeostasis (Chabot et al., 2016a), and the maximum metabolic rate (MMR), which is the highest achievable level of aerobic metabolism under the given environmental circumstances (Norin and Clark, 2016). The difference between MMR and SMR defines the aerobic scope and determines the maximum amount of energy available for movement, digestion, growth and reproduction (Fry, 1947; Claireaux and Lefrançois, 2007; McKenzie and Claireaux, 2010; Chabot et al., 2016b). Temperature inherently affects metabolic rates, and in turn, aerobic scope, of ectotherms through thermodynamic effects of biochemical functioning at the lower thermal range, and capacity limitations at the higher thermal range (Fry, 1947; Pörtner and Farrell, 2008; Schulte, 2015). Some animals can partially compensate for the effect of temperature change on metabolism, for instance by regulation of mitochondrial density and cardiorespiratory functions (Guderley, 2004; Seebacher et al., 2015a; Sandblom et al., 2016), which may aid to conserve as much aerobic scope as possible with temperature change (Tirsgaard et al., 2015). Animals with a high physiological performance over a broad environmental range may be readily compatible with novel environments (Sakai et al., 2001; Hellmann et al., 2008), and a high level of thermal compensation could thus be a common trait of invasive species.

Another valuable physiological trait is the upper thermal tolerance limit, referred to as the critical thermal maximum (CT_max_; Cowles and Bogert, 1944), as it is a proxy of a species’ resilience to thermal stress (Sunday et al., 2014) and correlates with latitudinal distribution of ectothermic animals (Mohseni et al., 2003; Sunday et al., 2011, 2012, 2014; Pinsky et al., 2019). CT_max_ depends on acclimation temperature, yet to a varying degree across species (Beitinger et al., 2000). A large increase in CT_max_ with increasing acclimation temperature is considered an enhanced capacity to handle thermal stress, such as during seasonal fluctuations or due to heat waves (Stillman, 2003; Williams et al., 2008; Shultz et al., 2016; Vinagre et al., 2016). As an example, the invasive lionfish (*Pterois* sp.) has a relatively large change in CT_max_ with temperature (Barker et al., 2018), compared with other tropical marine stenotherms (Stillman, 2003), presumably enabling the lionfish to cope well with environmental temperature increase and possibly facilitating their invasive success across a wide range of habitats.

Mobile ectotherms can actively choose a thermal environment that enables high physiological performance (temperature preference; Reynolds and Casterlin, 1979), including applying a reasonable thermal safety margin to detrimental temperatures (Kearney et al., 2009; Sunday et al., 2014). Investigations of behavioral thermoregulation of species, usually through their temperature preference (*T*_pref_) and avoidance (*T*_avoid_), can thus reveal how animals cope with thermal stress and, in turn, how behavioral thermoregulation may be coupled to physiological performance (Fry, 1947; Jobling, 1981; Pörtner and Knust, 2007). *T*_pref_ can vary with acclimation temperature, and the final temperature preferendum is defined as where *T*_pref_ equals the acclimation temperature (Reynolds and Casterlin, 1979; Jobling, 1981; Habary et al., 2016). In the invasive lionfish, *T*_pref_ is reported to be unaffected by acclimation temperature (Barker et al., 2018); such a lack of change in a trait after a change in the environment is indicative of a high level of phenotypic buffering (Reusch, 2014), in this case of behavioral thermoregulation, which could be a shared characteristic among invasive ectothermic species.

The round goby (*Neogobius melanostomus*) is one of the world's most impactful invasive fish species (Kornis et al., 2012; Ojaveer et al., 2015), and numerous negative effects have been reported in the wake of its invasion. For example, round gobies exert predation-driven alteration of entire benthic invertebrate communities (Lederer et al., 2006, 2008; Kipp and Ricciardi, 2012; Kipp et al., 2012; Barrett et al., 2017; Pennuto et al., 2018), predate on fish eggs, and exhibit superior competition for food, shelter and spawning grounds, thus posing a threat to native fishes (Lauer et al., 2004; Balshine et al., 2005; Fitzsimons et al., 2006; Karlson et al., 2007). Originally from the Ponto-Caspian region, the round goby has been introduced to, and become established in, a variety of temperate ecosystems, including the Laurentian Great Lakes in North America, rivers in central Europe, and the Baltic Sea (as far north as the Bothnian Sea and Bothnian Bay), and it continues to spread further in these areas (Jude et al., 1992; Sapota and Skora, 2005; Borcherding et al., 2011; Kornis et al., 2012; Azour et al., 2015; Puntila et al., 2018). The physico-chemical environment of the invaded areas differ markedly from the species' native range; the Baltic Sea has lower temperatures and is a brackish water habitat with different ionic composition than the Caspian and Black Seas, whereas the Great Lakes are large freshwater habitats (Dumont, 1998; Burns et al., 2005; Brown and Stepien, 2008; Schiewer, 2008; Kornis and van der Zanden, 2010; Kornis et al., 2012; Azour et al., 2015; Behrens et al., 2017). Thus, round gobies appear to have a wide tolerance for a variety of environmental parameters, making it an ideal model species for investigating physiological traits of an invasive species.

Here, we determined SMR, MMR, aerobic scope, CT_max_, *T*_pref_ and *T*_avoid_ of round goby acclimated to temperatures between 5 and 28°C. We hypothesized that physiological performance, evaluated as aerobic scope, would remain high across a broad range of temperatures. Due to the ability of round goby to invade a range of environments, we also expected that changes in acclimation temperature would lead to large changes in CT_max_, but small changes in *T*_pref_ and *T*_avoid_ as indicative of phenotypic buffering.

## MATERIALS AND METHODS

Holding and experimental protocols followed the principles of the three Rs (Cressey, 2015; Flecknell, 2002; Guhad, 2005), and were approved by the Danish Animal Experimentation Council (reference number: 2017-15-0201-01282).

### Fish and holding facilities

Round gobies [*Neogobius melanostomus* (Pallas 1814)] were obtained from fyke net fishers in the brackish waters of Guldborgsund (54°42′N, 11°51′E) and Karrebæksminde (55°10′N′ 11°39′E) in September and October 2018. Water temperature ranged between 10 and 15°C at the sampling time. The fish were transported for ∼1 h to the experimental facility at DTU Aqua, Lyngby, Denmark, in aerated and insulated transportation tanks. Upon arrival, the fish were treated in a 1:5000 formalin bath for 30 min to kill any ectoparasites, and subsequently tagged with passive integrated transponder tags (12×2 mm, 0.1 g) inserted into the body cavity of the fish. The fish were then given 3 weeks to recover from formalin treatment and tagging and adjust to the holding facility, during which time they were kept in 700 l holding tanks receiving filtered, recirculated and well-aerated water [dissolved oxygen (DO): 90–100% air saturation] at 10°C and a salinity of 10 (practical salinity unit; dimensionless). The inorganic nitrogen load was measured once a week (Testlab Marin; JBL, Neuhofen, Germany) and always stayed at acceptable levels ([NH_4_^+^] <0.05 mg l^−1^ at pH 8.1; [NO_2_^−^] <0.01 mg l^−1^; [NO_3_^−^] <20 mg l^−1^).

After the initial 3 weeks of holding, fish were distributed randomly across 24 compartments within six 700 l holding tanks with a maximum of eight fish per compartment. The temperature of the different compartments was gradually changed from 10°C to the specific target (acclimation) temperature (5, 10, 15, 20, 25 or 28°C) by 1°C day^−1^. The fish were then kept at their specific acclimation temperature for 3 weeks before experiments commenced. Acclimation to 30°C (rather than 28°C) was initially attempted; however, as mortality was seen during the acclimation phase at this temperature, 28°C was instead chosen as the highest acclimation temperature (using a different group of fish from the same collection batch, acclimated similarly to the other temperature treatments). Note that with the abovementioned holding and acclimation approach, we could statistically take into account tank effect (up to four replicates) within each acclimation temperature (up to six levels). To keep the effect of time to a minimum, all experiments were conducted within 4 weeks after acclimation to the target temperature.

Water temperature and water quality in the 10°C holding tank were maintained by a constant flow-through of 10°C system water. In the other holding tanks, the water temperature was controlled by either constantly heating with titanium rod heaters (AB Aqua Medic, Bissendorf, Germany; the 15–28°C treatments) or cooling with a chiller (TK 2000; Teco, Fornace Zarattini, Italy; the 5°C treatment) in combination with dosing in 10°C system water to counteract the temperature change and allow for precise temperature control. By controlling temperature in this way, a water exchange of 5–10% h^−1^ was ensured, which enabled maintenance of water quality at the levels mentioned above. Dosing of 10°C system water was controlled by programmable relays (PR 5714; PR Electronics, Rønde, Denmark) with a precision of 0.1°C.

The fish were fed 3 mm commercial pellets three times a week for the 5, 10 and 15°C treatments as in Behrens et al. (2017), and four times a week for the 20, 25 and 28°C treatments. The higher feeding rates at the higher temperatures were applied to account for an increase in basal energetic demand with increasing temperature, and to prevent starvation. To avoid elevated metabolism due to digestion (Chabot et al., 2016b), feeding was withheld for 5 days for the 5°C treatment, 3 days for the 10, 15 and 20°C treatments, and 2 days for the 25 and 28°C treatments prior to experimentation (Secor, 2009). The shorter fasting at higher temperatures were applied as digestion is faster at increasing temperatures in fish (Frisk et al., 2013).

The lighting was kept dim and on a 9 h:15 h light:dark photoperiod throughout the study period.

### Metabolic rates

We measured oxygen consumption rate (*Ṁ*_O_2__) as a proxy for aerobic metabolic rate (Nelson, 2016) after acclimation to 5, 10, 15, 20, 25 or 28°C. *Ṁ*_O_2__ was measured on individual fish (*N*=78, 10–16 fish per temperature) with a body mass (*M*_b_) of 49±2 g (mean±s.e.m.) (Table 1) using intermittent-closed respirometry (Steffensen, 1989; Clark et al., 2013; Svendsen et al., 2016). Four cylindrical, acrylic, resting respirometers with total volumes of 1.136–1.153 l were run in parallel. Each respirometer consisted of a recirculation loop of PVC tubing connecting each end of the respirometer via an in-line pump (Eheim Universal 1046; Eheim & Co. KG, Deizisau, Germany), which mixed the water in the respirometer and moved it past an optical oxygen probe connected to a 4-channel oxygen meter (FireStingO_2_; PyroScience, Aachen, Germany). A second Eheim Universal 1046 pump was used to intermittently flush fully aerated water through the respirometers from an 80 l ambient tank, in which the respirometers were immersed, draining back into the ambient tank through an overflow PVC tube exiting the respirometer at the end opposite the flush inlet. The flow of the pumps was reduced to prevent the fish from swimming against the water current. The ambient tank was connected to a 70 l pump sump in which the water was aerated and temperature controlled (with a precision of 0.1°C in a similar manner as in the acclimation tanks), ensuring a 30% water turnover each hour. To minimize disturbance of the fish, the set-up was shielded by a tarpaulin. The DO level (in % air saturation) inside the respirometers was measured in the recirculation loop at 1 Hz and logged on a PC. The oxygen sensors were calibrated at the experimental temperatures to 100% DO in fully aerated water, and to 0% DO using a 10 g l^−1^ NaSO_2_ solution with an equal amount of boric acid. The oxygen recordings were temperature compensated by the oxygen meter. The *Ṁ*_O_2__ experiments were automated with a PC using AquaResp software (www.aquaresp.com), which controlled the flush pump with a mechanical relay through a USB interface (USB-SwitchC; Cleware, Hollingstedt, Germany). AquaResp also acquired the data and calculated the slope for the fractional change in DO over time for each intermittent measuring period by linear regression.

**Table 1.**
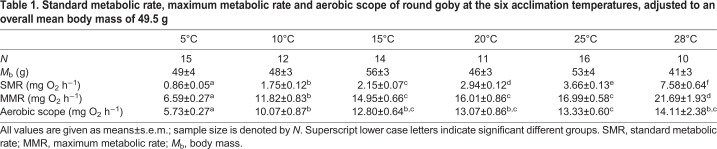
**Standard metabolic rate, maximum metabolic rate and aerobic scope of round goby at the six acclimation temperatures, adjusted to an overall mean body mass of 49.5** **g**

All trials started around noon; the fish were weighed and subjected to an exhaustive chase protocol in order to elicit their maximum metabolic rate (MMR) (Reidy et al., 1995; Norin and Clark, 2016; Behrens et al., 2017). The protocol consisted of chasing the fish for 2 min in a 60 cm diameter bucket containing water at the experimental temperature, after which the fish were no longer responsive to chasing. After the chase, the fish were air exposed for 1 min (Clark et al., 2012) and immediately thereafter placed in the respirometers, which were sealed within 1 min after the end of air exposure. After initial wait times of between 45 and 120 s to allow for full mixing of the water in the respirometer before measurements (the time being dependent on the experimental temperature), the first *Ṁ*_O_2__ measurement was determined over 180–390 s. The measuring and wait times were longer at the lower temperatures to obtain decent drops in DO (Svendsen et al., 2016).

After the chase protocol, the measuring times were altered to between 210 and 1140 s depending on temperature, and then the fish were left undisturbed for 20–22 h to measure their SMR. The flush time was always 240 s and ensured>99% exchange of the water inside the respirometers (Steffensen, 1989). After SMR determinations, fish were removed from the respirometers and background (microbial) respiration measured in the empty chambers using measuring times of 1800–3600 s and initial wait times of 200–400 s, the length of the periods decreasing with increasing temperature (Svendsen et al., 2016).

*Ṁ*_O_2__, corrected for background respiration (BR), was calculated according to Svendsen et al. (2016):(1)
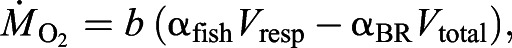


where *b* is the oxygen solubility in mg O_2_ l^−1^ at the given temperature, salinity and atmospheric pressure, α_fish_ is the fractional change in DO over time (h^−1^), *V*_resp_ is the respirometer volume (l) with the fish in the respirometer, α_BR_ is the decrease in fractional change in DO over time of the BR measurement, and *V*_total_ is the total volume (l) of the respirometer without the fish. *V*_resp_ was calculated as *V*_total_ minus the volume of the fish (*V*_fish_), where a density of 1 g ml^−1^ was assumed for the fish.

The *Ṁ*_O_2__ measurements with linear regression of α of *r*^2^<0.95 were considered outliers and excluded from data processing (Svendsen et al., 2016). The first measurement during an experimental trial (i.e. immediately after the chase protocol) was taken as the MMR of the fish. SMR was determined by the double Gaussian distribution fit method (Fig. S1; Steffensen et al., 1994; Chabot et al., 2016a).

### Critical thermal maximum

CT_max_ was determined as the temperature where the fish lost equilibrium and tipped onto a side or belly-up position during an acute heating event. The experiment was performed on fish with *M*_b_ of 41±2 g and acclimated to either 10 (*N*=12), 20 (*N*=10) or 28°C (*N*=11). The rate of acute temperature increase was 2.0°C h^−1^ to keep the increment at an ecologically relevant rate (2.0°C h^−1^ is approximately the maximum rate of daily change occurring in different aquatic habitats in nature; e.g. Rodnick et al., 2004; Loong et al., 2005; Richards, 2011; Vinagre et al., 2016) and to minimize the effect of lag in heat exchange between the environment and the core temperature of fish of the size used in the present study (Becker and Genoway, 1979). Fish were tested in groups of three to four, as in Morgan et al. (2018), providing three experimental replicates per temperature treatment. The set-up consisted of an 80 l arena connected to a 70 l pump sump in which the water was aerated and temperature regulated. Temperature was measured in the arena at 1 Hz and logged onto a PC (FireStingO_2_ temperature sensor and Oxygen Logger software; PyroScience). A linear temperature increase of 2.0°C h^−1^ was obtained with titanium rod heaters (AB Aqua Medic, Bissendorf, Germany). The fish were weighed and placed in the arena at their acclimation temperature (i.e. 10, 20 or 28°C) in the afternoon around 15:00 h and were allowed 12–15 h to settle in the set-up before the experimental trial commenced. The CT_max_ trials were thus all conducted at the same time of day. As loss of equilibrium is not always immediately apparent for benthic fish such as round goby, non-moving fish were gently poked with a stick every 5 min at temperatures above 31°C (31°C was determined in pilot experiments as being below, but close to, CT_max_). Immediately after reaching CT_max_, the fish were transferred to their acclimation temperature for recovery. To minimize disturbance of the fish, the set-up was shielded by a tarpaulin and the arena was monitored with a USB video camera (Microsoft LifeCam HD-3000; Microsoft, Redmond, WA, USA) via a PC.

### Temperature preference and avoidance

*T*_pref_ and *T*_avoid_ were assessed in fish with *M*_b_ of 53±3 g acclimated to either 10°C (*N*=12) or 20°C (*N*=13). The experiments were conducted on individual fish in a shuttle tank (Loligo Systems, Viborg, Denmark; cf. Schurmann et al., 1991; Macnaughton et al., 2018) consisting of two 40 cm wide, circular compartments inter-connected by a 10 cm×7 cm passage. The water level was approximately 10 cm. Each compartment was connected to an adjacent 10 l mixing tank in which the water was aerated and the temperature measured. Temperature was regulated by pumping water from the mixing tanks through heat exchangers in either a heating or a cooling bath (40 and 5°C, respectively). The temperature regulation pumps were controlled and activated from a PC with ShuttleSoft software (Loligo Systems) through a mechanical relay with a USB interface (DAQ-M; Loligo Systems). The shuttle tank had a translucent bottom and was illuminated from underneath with infrared light (850 nm) to enhance the contrast between the fish and its background. The set-up was monitored with an infrared-sensitive USB 2.0 camera (UI-1640SE; IDS Imaging Development Systems, Obersulm, Germany). To minimize disturbance, the set-up was shielded by a tarpaulin. The ShuttleSoft software logged the temperature in the two mixing tanks through the DAQ-M unit, and tracked the position and activity of the fish in real time via the USB camera.

On the day of an experiment, an individual fish was placed in the shuttle tank. The water temperature in the shuttle tank was kept constant at the acclimation temperature of the fish [±0.5°C (s.d.)] for the first 2 h of the experiment to allow the fish to settle in the shuttle tank. After the first 2 h, the system was set to dynamically regulate the temperature according to the position of the fish. More specifically, the presence of the fish on one side of the shuttle tank initiated an increase in the overall temperature of the system, whereas the presence of the fish on the other side initiated a decrease in temperature. The temperature changes occurred at rates of 0.3°C min^−1^, with a constant temperature difference of 2.0°C between the two compartments of the shuttle tank. This meant that the fish would sense an immediate, but small, temperature difference when moving between compartments, and thus be able to regulate its core temperature by shuttling back and forth between the two sides of the shuttle tank. All trials started around noon and lasted ∼23 h.

For each experiment, the preferred temperature was defined as the median of all the temperatures the fish had experienced during a trial, after excluding the first 2 h where the fish was settling. The median is considered a robust measure of *T*_pref_ when the fish have occupied a broad range of temperatures or when the temperature occurrence is different from that of a normal distribution (Schurmann et al., 1991). However, using the median as estimate of *T*_pref_ is potentially affected by any initial temperature change before the fish settles around its preference temperature, depending on how long it takes the fish to reach this (defined as the experimental stabilization time). To test the robustness of the chosen method for temperature preference determination (overall median) as well as the experimental stabilization time, a ‘hockey stick’ regression was fitted to the data for temperature over time with the software R (https://www.r-project.org/; Fig. S2). The hockey stick approach fits any initial change in temperature to the sloping part of the regression, whereas the horizontal part of the regression is fitted to the part of the experiment where temperature is stable. The stable part of the regression was regarded as a secondary measure of temperature preference (*T*_pref,sec_), and the experimental stabilization time was defined as the intersect between the two regression lines. It should be noted that although the hockey stick method estimates a temperature preference regardless of any initial change in temperature, it does so using the linear least squares method and, thus, fits data that are evenly distributed around the mean well, but is less accurate if the fish has chosen a skewed distribution of temperatures during the experiment. The first and third quartiles of the distribution of temperatures experienced by the fish were calculated to illustrate the variation in experienced temperatures around *T*_pref_, and these were defined as the lower and upper avoidance temperatures (*T*_avoid,low_ and *T*_avoid,up_, respectively). The upper thermal safety margin is normally considered to be the difference between a species CT_max_ and the temperatures it regularly experiences in the wild (Sunday et al., 2014), and we therefore defined it as CT_max_−*T*_avoid,up_.

### Data analyses and statistics

All data were analysed in R v.4.0.1 (https://www.r-project.org/). Linear mixed-effects models (lme4 package; Bates et al., 2015) were constructed to assess the effects of acclimation temperature on the measured traits. The models had log_10_-tranformed SMR, log_10_-transformed MMR, CT_max_, *T*_pref_, *T*_avoid,low_ or *T*_avoid,up_ as response variables. All models had acclimation temperature, log_10_-transformed *M*_b_ and sex as fixed effects, while compartment (nested within acclimation temperature) was included as a random effect. Statistical significance (*P*-values) was evaluated from the lmerTest package (Kuznetsova et al., 2017). For all models, model selection was performed using maximum likelihood estimation, taking out least significant variables sequentially. Variables were kept in the model if their removal resulted in significant (*P*<0.05) likelihood ratio tests. The assumptions of homoscedasticity and normality of residuals were examined by visual inspection of residual-fit plots. For SMR, the plot of empirical versus theoretical quantiles (normal Q–Q plot) indicated possible deviation from normality, which was followed up with a Shapiro–Wilk normality test on the model residuals. This test indicated normality at *P*=0.07.

As fish body mass had a significant effect on SMR and MMR, the *Ṁ*_O_2__ of all fish (in units of mg O_2_ h^−1^) was adjusted to a common body mass (*M*_b,adjusted)_ of 49.5 g (the overall mean *M*_b_) using the parameter estimate for *M*_b_ (the scaling exponent, *b*) from the model for either SMR or MMR, according to:(2)
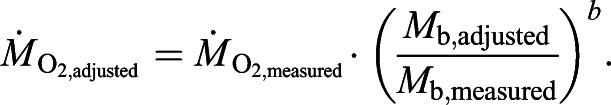
Aerobic scope was calculated as MMR minus SMR using the *M*_b_-adjusted values. The adjusted *Ṁ*_O_2_ _values were used for further analyses and graphical presentations. Duncan's *post hoc* tests were used to identify differences between treatment (acclimation) groups.

The factor by which SMR, MMR and aerobic scope changed with a 10°C temperature change (*Q*_10_) was calculated from the slope of a linear regression on semi-log transformed data. Furthermore, *Q*_10_ values were calculated between each consecutive acclimation temperature by the van't Hoff equation:(3)
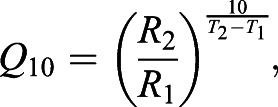
where *R*_1_ and *R*_2_ are *Ṁ*_O_2__ at the lower temperature (*T*_1_) and the higher temperature (*T*_2_), respectively.

To visually evaluate trends in the data, the data for SMR as a function of temperature were fitted with a two-parameter exponential function, while the MMR and aerobic scope data were fitted with a sigmoid (logistic) function.

The experimental stabilization time was compared between the two acclimation temperatures with a Mann–Whitney *U*-test, as the data were not normally distributed. The temperature preference calculated with the median method (*T*_pref_) was compared with *T*_pref,sec_ with a Student's *t*-test.

## RESULTS

### Oxygen consumption rates

**Fig. 1.**
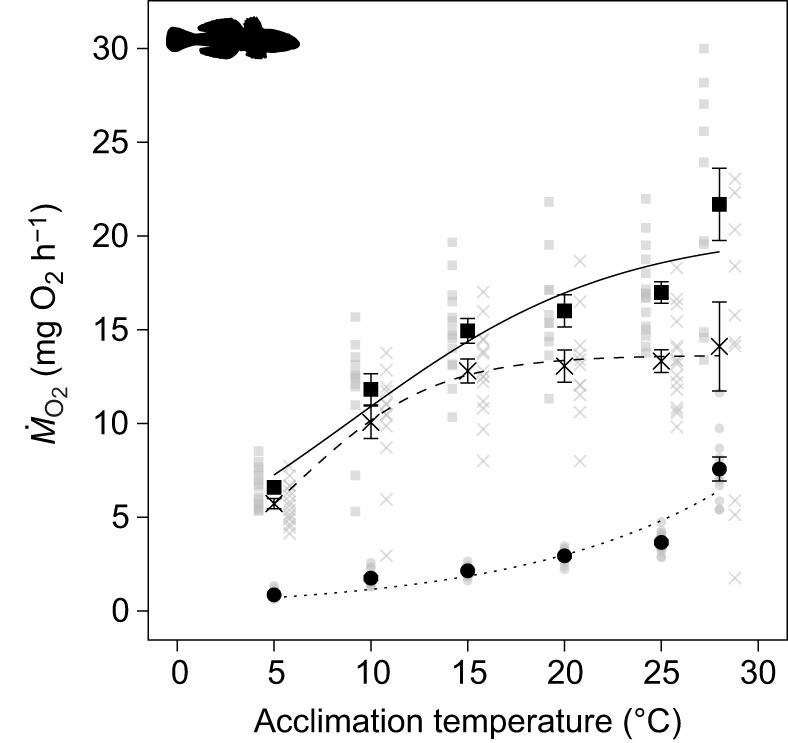
**The effect of acclimation temperature (*T*)** **on metabolic rates (*Ṁ*_O_2__) of round goby.** Circles represent standard metabolic rate (SMR) and the dotted line is a fitted exponential regression (*Ṁ*_O_2__=0.444[0.090]*e*^(0.095[0.008]×*T*)^). Squares represent maximum metabolic rate (MMR) and the continuous line is a fitted logistic (sigmoid) regression (*Ṁ*_O_2__=20.342[1.597]/ (1+*e*^(−0.147[0.036]×(*T* – 9.011[1.422]))^)). Crosses represent aerobic scope (MMR minus SMR) and the dashed line is a fitted logistic regression (*Ṁ*_O_2__=13.645[0.635]/(1+*e*^(−0.280[0.085] (*T* – 6.176[0.871]))^)). Values in square brackets in the regression equations are s.e.m. of the regression coefficients. Black symbols with error bars are means±s.e.m. and gray symbols are individual *Ṁ*_O_2__ measurements. Individual data points for MMR and aerobic scope have been offset slightly around the actual experimental temperature to prevent overlap.

The scaling exponent for SMR was 1.047 and was not affected by acclimation temperature (body mass–temperature interaction: *t*_60.79_=−1.335, *P*=0.187). Acclimation temperature had a positive effect on SMR (*F*_5,71_=191.21, *P*<0.0001), with SMR increasing significantly between each increment in acclimation temperature (Fig. 1; Table 1). The scaling exponent for MMR was 0.813 and was also not affected by acclimation temperature (body mass–temperature interaction: *t*_63.30_=−0.058, *P*=0.954). Acclimation temperature also had a positive effect on MMR (*F*_5,70_=54.18, *P*<0.0001), with post hoc tests showing an increase in MMR from 5 to 15°C, a plateau from 15 to 25°C, and a final increase again at 28°C (Fig. 1; Table 1). This pattern was mirrored in aerobic scope, which was also affected positively by acclimation temperature (*F*_5,72_=10.74, *P*<0.0001) but had a plateau extending all the way from 15 to 28°C (Fig. 1; Table 1). Interestingly, sex had a significant effect on MMR (*P*=0.041), and MMR only, with males having an overall 26.4% higher MMR than females after accounting for variation in body mass. However, out of the 78 individuals used in the *Ṁ*_O_2__ experiment, only 11 were female, and females were not represented in all acclimation temperature groups. Consequently, the data for both sexes are pooled in Fig. 1 and Table 1.

The *Q*_10_ values for SMR ranged between 1.50 and 1.88 in the middle range of the acclimation temperatures (between 10 and 25°C), yet were substantially augmented at both temperature extremes, that is, from 5 to 10°C and 25 to 28°C (values of 4.13 and 11.31, respectively; Table 2). The *Q*_10_ values for MMR were also lowest in the middle range of acclimation temperatures (values between 1.13 and 1.60), and markedly higher at the temperature extremes (values of 3.21 and 2.26; Table 2). For aerobic scope, *Q*_10_ was highest at the lower temperature extreme (3.09) and converged to values between 1.04 and 1.62 at temperatures above 10°C (Table 2). The overall *Q*_10_ values ranged between 1.36 and 2.18 and were highest for SMR and lowest for aerobic scope (Table 2).

**Table 2.**
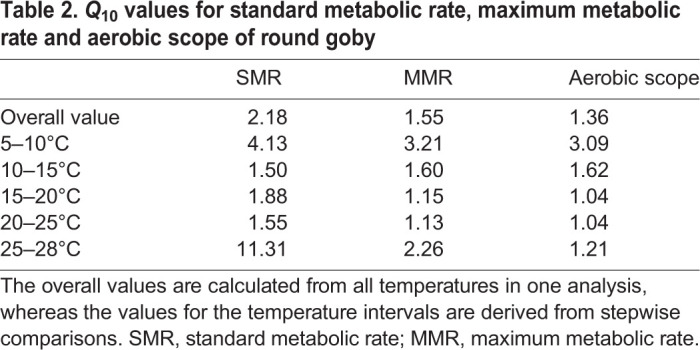
***Q*_10_ values for standard metabolic rate, maximum metabolic rate and aerobic scope of round goby**

### Critical thermal maximum

**Fig. 2.**
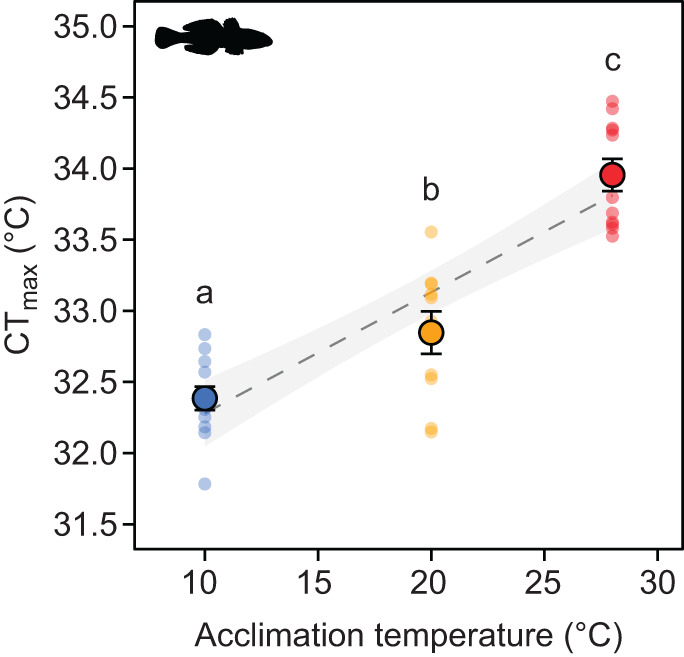
**The effect of acclimation temperature (*T*) on critical thermal maximum (CT_max_) of round goby.** Large circles with error bars are means±s.e.m. and smaller circles are individual data points. Different lower case letters within the panel indicate statistical differences (*P*<0.05) between acclimation groups. The dashed line is a linear regression (CT_max_=31.43[0.20]+0.09[0.01]×*T*; *r*^2^=0.71, *P*<0.0001) surrounded by its 95% confidence band in light gray. Values in square brackets in the regression equation are s.e.m. of the regression coefficients.

CT_max_ increased significantly with acclimation temperature (*F*_1,31_=76.30, *P*<0.0001), from 32.4±0.1°C for fish acclimated to 10°C to 32.8±0.1 and 34.0±0.1°C (means±s.e.m.) for fish acclimated to 20 and 28°C, respectively (Fig. 2). Overall, CT_max_ increased by 0.09°C per 1°C increase in acclimation temperature, with the highest increase in CT_max_ occurring at an acclimation temperature of 28°C relative to both the 10 and 20°C acclimation temperatures (Fig. 2). Although we attempted to preclude mortalities in non-moving fish by gently poking them with a small stick every 5 min when temperatures increased above 31°C, we still encountered 33% mortality after the CT_max_ experiments in the 10°C acclimation group, 20% in the 20°C acclimation group, and 45% in the 28°C acclimation group.

### Temperature preference and avoidance

The experimental stabilization times for the temperature preference experiments were significantly different (*P*=0.047) between fish acclimated to 10°C (2.8±0.9 h) and fish acclimated to 20°C (1.0±0.4 h). *T*_pref_ and *T*_pref,sec_ were not significantly different (Student's *t*-test, *P*=0.835).

**Fig. 3.**
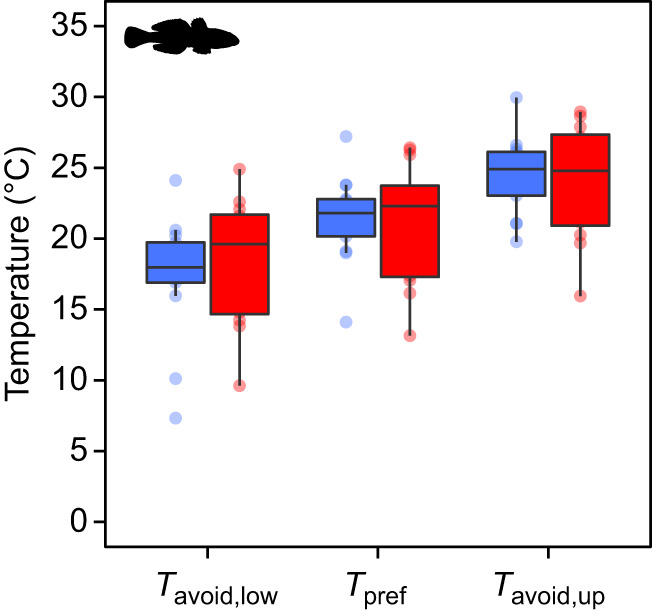
**The effect of acclimation temperature on preference and avoidance temperature of round goby.** Lower avoidance temperature (*T*_avoid,low_), preference temperature (*T*_pref_) and upper avoidance temperature (*T*_avoid,up_) were determined in fish acclimated to 10°C (blue) and 20°C (red). Horizontal lines on boxes represent first, second and third quartiles, while whiskers extend to individual data points (circles) that are within 1.5 times the interquartile range. None of the metrics was significantly affected by acclimation temperature (*P*>0.62).

Acclimation temperature (10 and 20°C) did not significantly affect *T*_pref_ (*F*_1,23_=0.05, *P*=0.825), which was 21.2±0.7°C (mean±s.e.m.) for both groups combined (Fig. 3). *T*_avoid,low_ was also not significantly affected by acclimation temperature (*F*_1,23_=0.25, *P*=0.624), the mean for both groups being 17.8±0.9°C. Likewise, *T*_avoid,up_ was not affected by temperature (*F*_1,23_=0.07, *P*=0.793), the mean being 24.3±0.7°C for both groups combined. The thermal safety margins were 8.1 and 8.5°C at acclimation temperatures of 10 and 20°C, respectively.

## DISCUSSION

We found that the invasive round goby maintained unperturbed physiological performance from 15 to 28°C, evidenced by the maintenance of a relatively high aerobic scope at these temperatures. Maintaining high physiological performance over broad environmental ranges can make species more readily compatible with rapidly changing environments and give them a competitive advantage, and may thus enable species to become invasive in a range of different habitats (Sakai et al., 2001; Hellmann et al., 2008). The *Q*_10_ values for aerobic scope were close to 1 from 15 to 28°C, indicating almost complete thermal compensation for this important physiological trait within a wide temperature range (Seebacher et al., 2015a). When environmental changes induce stressful conditions at the edge of tolerance ranges, a high level of thermal compensation of physiological performance is evolutionarily favorable (Reusch, 2014) and may provide a competitive advantage for species experiencing increases in environmental temperature, as well as in the frequency and amplitude of thermal fluctuations as results of climate change (IPCC, 2013).

The *Q*_10_ values of SMR were below 2 at the intermediate temperatures (10–25°C), showing high acclimation capacity of the round goby in this temperature range (Sandblom et al., 2014). However, SMR was substantially augmented at 28°C compared with 25°C, resulting in a very high *Q*_10_ of 11.31 for this temperature range. This suggests a profound loss of capacity for thermal acclimation of SMR at the highest temperature, which was close to the upper lethal temperature under chronic exposure, as evident by the increased mortality we observed during our initial attempt to acclimate the fish to 30°C. The abrupt increase in SMR from 25 to 28°C could be caused by an increasing inability to defend membrane integrity at high temperatures: changes in cell membrane phospholipid composition with changing temperature is one of the most profound physiological changes in ectothermic animals (Hochachka and Somero, 2002), and altered membrane phospholipid composition affects energetically demanding processes such as Na^+^–K^+^-ATPase activity and restoring the mitochondrial proton gradient in the face of increased leakage across more fluid membranes (Hulbert and Else, 2005). The loss of capacity for thermal compensation of SMR at 28°C could also be due to an onset of other energetically demanding processes to cope with high temperature, such as production of heat shock proteins and anti-oxidant enzymes (Iwama et al., 1999; Sørensen et al., 2003; Heise et al., 2006; Loughland and Seebacher, 2020). Heat shock proteins and anti-oxidant enzymes stabilize altered proteins and alleviate protein degradation in response to thermal stress (Kiang and Tsokos, 1998; Basu et al., 2002; Heise et al., 2006). While this is speculative, as we did not measure cell membrane processes, oxidative stress responses, heat shock proteins or enzyme functionality alongside SMR, it could be a target for future studies. Regardless of the mechanisms at play, the observed increase in MMR at 28°C nonetheless helped to conserve aerobic scope in the face of an increasing SMR at this temperature. MMR represents the maximum rate at which aerobic physiological processes can occur (McKenzie and Claireaux, 2010; Norin and Clark, 2016), and it is possible that an onset of cellular and rate protection processes at 28°C (indicated by the profoundly elevated SMR at this temperature) may have increased the functionality of physiological processes to a level that also caused a substantial increase (improvement) in MMR at 28°C.

At the other end of the thermal range, SMR decreased markedly at 5°C, yielding a high *Q*_10_ value (4.13) compared with SMR at 10°C. Such a decrease in SMR towards the lower range of the thermal envelope has previously been shown in American eel (*Anguilla rostrata*) (Walsh et al., 1983), European eel (*Anguilla anguilla*) (Methling, 2013) and Atlantic cod (*Gadus morhua*) (Tirsgaard et al., 2015), and has been interpreted as a means to preserve as much aerobic scope as possible during depression of MMR at lower temperatures (Tirsgaard et al., 2015). Aerobic scope was, however, still diminished at temperatures at and below 10°C in the present study, indicating that these temperatures were unfavorable for the aerobic performance of round goby. This may restrict distribution at colder temperatures (Wolfe et al., 2020) and, in turn, limit secondary range expansion into colder parts of newly invaded areas. Outside its native range, the round goby has presumably reached its northernmost boundary in the Great Lakes (Kornis et al., 2012) and, similarly, has not established in the most north-eastern and coldest areas of the Baltic Sea (Puntila et al., 2018). However, as global temperatures continue to increase, this highly invasive species may begin to also colonize these northern environments from which it is currently excluded.

A high intra-specific divergence in physiological performance at environmental extremes has been shown to relate to a generalist–specialist trade-off between acclimation capacity and overall performance in the invasive mosquitofish (*Gambusia holbrooki*) (Seebacher et al., 2015b). In the present study, we found a pronounced inter-individual variation in aerobic performance of round goby at the highest temperature (28°C): some individuals displayed their highest MMR and aerobic scope at this temperature, while the aerobic performance of others (three out of 10 individuals) was negatively affected. A comparable phenomenon has been observed for round goby acclimated to salinities varying from freshwater to nearly full-strength seawater: some individuals were able to maintain unperturbed blood plasma osmolality levels at the highest salinities (25 and 30), while others partially conformed to the ambient salinity, causing higher and sub-optimal blood plasma osmolality (Behrens et al., 2017). Such phenotypic diversity among individuals has been suggested as a reason for the invasive success of mosquitofish (Seebacher et al., 2015b), which may also apply to round goby, both with respect to temperature (present study) and salinity (Behrens et al., 2017).

The CT_max_ observed in the present study (32.2–34.0°C) aligns with earlier findings in freshwater round goby from the Great Lakes (CT_max_ of 33.4±0.3°C; Cross and Rawding, 2009). While the study by Cross and Rawding (2009) used only one acclimation temperature (15°C), we show here that CT_max_ of round goby increased with acclimation temperature at a rate of 0.09°C per 1°C. This change in CT_max_ with change in acclimation temperature of round goby is around fourfold lower than the mean rate of change in CT_max_ reported for 20 species of freshwater fish (0.41°C per 1°C change in acclimation temperature; Beitinger et al., 2000), and markedly lower than the rate of change in CT_max_ of the invasive lionfish (0.5°C per 1°C; Barker et al., 2018). It should be noted that our heating rate during CT_max_ experiments was lower than that in Beitinger et al. (2000) and Barker et al. (2018), which may have led to lower estimates of CT_max_ (Becker and Genoway, 1979). To what extent this could have affected changes in CT_max_ with changing acclimation temperature remains unknown.

Although we expected to find a large change in CT_max_ with changing acclimation temperature in round goby as an indication of resilience to thermal stress, this was not the case. Instead, we found an upper thermal safety margin of 8.1 and 8.5°C (i.e. the difference between CT_max_ and *T*_avoid_), which is relatively large compared with other species (Vinagre et al., 2016). These results suggest that an invasive species may obtain resilience to thermal stress through behavioral thermoregulation, and not only through a change in thermal tolerance (CT_max_) with changing acclimation temperature, which can make it less vulnerable to global warming (Vinagre et al., 2016).

Phenotypic buffering, i.e. maintaining the same level of a trait after a change in the environment, is evolutionarily favorable when environmental changes induce stressful conditions at the edge of tolerance ranges (Reusch, 2014). The lack of change in round goby *T*_pref_ and *T*_avoid_ with increasing acclimation temperature indicates a high level of phenotypic buffering for acute behavioral thermoregulation, and may give round goby a competitive advantage in fluctuating environments (Loughland and Seebacher, 2020). Interestingly, *T*_pref_ of the invasive lionfish also did not change with acclimation temperature (Barker et al., 2018), adding to the notion that high levels of phenotypic buffering of behavioral thermoregulation may be advantageous for species introduced to novel environments, and could potentially be a common characteristic of invasive species in general. As climate change is not only resulting in an overall warmer world, but also alters the frequency and amplitude of temperature fluctuations (IPCC, 2013), a high level of phenotypic buffering for behavioral thermoregulation in invasive species may only increase their invasive potential in the future.

It has been hypothesized that *T*_pref_ should coincide with the temperature where overall performance (e.g. growth) is highest, and the temperature where aerobic scope is maximized (Pörtner and Knust, 2007; Pörtner et al., 2017). This theory assumes that the temperature preference of a species is determined by limitations in meeting organismal oxygen demands at high temperature, which restricts physiological performance and is aptly named the oxygen- and capacity-limited thermal tolerance (OCLTT) theory. Some empirical evidence supports the OCLTT theory (Reynolds and Casterlin, 1979; Jobling, 1981; Habary et al., 2016; Christensen et al., 2020) while other evidence does not (Clark et al., 2013; Gräns et al., 2014; Norin et al., 2014; Jutfelt et al., 2018). For the round goby, the preferred temperature (21.2°C) fell in the middle of the temperature range that enabled the fish to achieve a high aerobic scope (15–28°C), but was lower than the temperature where the fish achieved the numerically highest aerobic scope (28°C). These findings fit well with the idea that ‘suboptimal is optimal’ (cf. Jensen's inequality; Martin and Huey, 2008) as it allows the fish to maintain a thermal safety margin, so that any unexpected, sudden elevations in temperature will not reduce its aerobic scope to zero. The OCLTT theory generally assumes that aerobic scope should decrease at high temperatures, yet this evidently does not apply to round goby. Although speculative, it is possible that species with an uncompromised aerobic scope at high temperatures may be superior in competition with species whose aerobic scope decreases, and that these species may have a larger invasive potential if introduced into ecosystems containing species that are oxygen and capacity limited at their thermal extremes.

Extrapolation of species distributions to future environmental conditions using mechanistic species distribution models are more robust when parameterized with physiological and behavioral traits (Peterson, 2003; Kearney and Porter, 2009; Buckley et al., 2010; Evans et al., 2015). For ectotherms in particular, the use of thermal performance, thermal tolerance and behavioral thermoregulation has proven useful in predicting geographical distributions (Pörtner and Knust, 2007; Pörtner and Farrell, 2008; Eliason et al., 2011; Jørgensen et al., 2012; Sunday et al., 2012; McKenzie et al., 2016; Pinsky et al., 2019). In comparison with the physiological and behavioral traits of native species (Zarnetske et al., 2012; Blois et al., 2013; Milazzo et al., 2013), mechanistic models may aid in predicting areas where potential introduction of known invasive species are likely to have adverse effects on native species, as well as areas where the introduced species will pose little or no threat to the ecosystem, both now and in a warmer future (Kearney et al., 2009; Walther et al., 2009; Woodin et al., 2013; Marras et al., 2015). Our results suggest that high levels of phenotypic buffering of metabolism, thermal tolerance and behavioral thermoregulation could be central traits of invasive species, which could be incorporated into such models.
